# Metabolomic and transcriptomic analyses reveal a MYB gene, *CsAN1*, involved in anthocyanins accumulation separation in F1 between ‘Zijuan’ (*Camellia sinensis var. assamica*) and ‘Fudingdabaicha’ (*C. sinensis var. sinensis*) tea plants

**DOI:** 10.3389/fpls.2022.1008588

**Published:** 2022-09-21

**Authors:** Feiyi Huang, Jihua Duan, Yu Lei, Yankai Kang, Yi Luo, Yingyu Chen, Ding Ding, Saijun Li

**Affiliations:** Tea Research Institute, Hunan Academy of Agricultural Sciences/National Centre for Tea Improvement, Hunan Branch/Hunan Tea Variety and Seedling Engineering Technology Research Center, Changsha, China

**Keywords:** purple-leaf tea, anthocyanins, metabolomics, *CsANS1*, MYB

## Abstract

‘Zijuan’ (*Camellia sinensis* var. *assamica*), a somatic mutant with purple foliage and stem selected from the Yunnan Daye cultivar, has been well developed owing to abnormal pattern of anthocyanin accumulation. However, the genetic basis for the specific accumulation of phloem glycosides is not clear. Tea plants are self-incompatible, so parents with large differences in foliage color were used for crosses to investigate the genetic mechanism of anthocyanins. In this study, ‘Zijuan’ and green foliage cultivar ‘Fudingdabaicha’ (*C. sinensis* var. *sinensis*) were used as female and male parents, respectively, to generated F1 hybrid progenies with various anthocyanin contents. In order to decipher the genetic rules of anthocyanins accumulation, we performed widely targeted metabolic and transcriptomic profiling. The results showed that cyanidin-3-*O*-galactoside, delphinidin-3-*O*-galactoside and petunidin-3-*O*-galactoside were the major types of anthocyanins and factors directly led to the color variation between parents and F1 plants. Transcriptomic analyses suggested the significant up-regulation of anthocyanidin synthase gene (*CsANS1*) and *CsAN1*, a MYB family gene positively regulated the expression of *CsANS1*, in anthocyanin-rich tea plants. Furthermore, the deletion mutation of *CsAN1* was found by cloning and alignment in anthocyanin-lacking cultivars. Taken together, the function deficiency of *CsAN1* is predominantly responsible for the inability of anthocyanins accumulation, and this trait is heritable in progenies through hybridization. The present study elucidated the molecular basis of leaf purple trait formation in ‘zijuan’ and ‘Fudingdabaicha’ and their F1 plants, which helps to elucidate the genetic mechanism of leaf anthocyanin accumulation regulation in tea plants, and the results provide a research reference for the selection and breeding of high anthocyanin type tea varieties.

## Introduction

Tea plants (*Camellia sinensis*) from the family Theaceae are a perennial evergreen woody plant species that is cultivated widely in the tropical and subtropical district worldwide, including mainly China, Japan, and Kenya (Zhao et al., 2020). Tea, generally processed from the leaf of tea plants, provides abundant secondary metabolites which enrich our taste and improve human health as the world’s most prevalent beverage. The tasty flavors and different health-promoting functions of tea mainly owe to a great deal of bioactive compounds (Koch et al., 2019). Among these, the most characteristic metabolites are caffeine, theanine, flavonoids, and some volatiles (Turkozu and Sanlier, 2017). Among the abundant beneficial metabolites in tea plants, flavonoids, belonging to the phenolic compounds, has been paid much more attentions because of being nutraceutical and traditional medicine for human in recent years. Generally, flavonoids include anthocyanins, flavones, flavonoids, isoflavones, flavanols, flavonols, and some derivatives (e.g., catechins) (Nabavi et al., 2020). Anthocyanins are the primary pigments that determine the coloration in human edible and ornamental plants, such as many tropical and subtropical fruits, purple crops, and varicolored flowers (Tirumalai et al., 2019; Li et al., 2020b; Fu et al., 2021; Yi et al., 2021). For the plants, anthocyanins play significant roles for attracting pollinators and protecting plants against various abiotic stresses (Zhou et al., 2014). In addition, numerous studies and observations of animal models and human clinical trials show that anthocyanins have antioxidative and antimicrobial activities, improve neurological and visual health, and prevent various chronic diseases (Khoo et al., 2017). Purple-leaf tea plants, a novel germplasm of tea plants with rich anthocyanins accumulation (e.g., cv. ‘Baitang purple tea’), has been well developed owing to its attractive, unique color and pharmacological action responsible for prevention of some chronic diseases (Rothenberg et al., 2019; Tang et al., 2021). Pharmacological result shows that the purple-leaf tea extract can restrain the cancer proliferation by interrupting cell cycle progression and inducing apoptotic death (Hsu et al., 2012). Moreover, some valuable studies suggested that the purple-leaf tea extract plays a positive role in physiological activity including anti-trypanosome, cerebral antioxidants, and anti-obesity activities (Rashid et al., 2014a; Rashid et al., 2014b; Hayat et al., 2015; Hiroshi et al., 2015). Hence, the anthocyanins-rich tea plant germplasms have been paid much more attentions due to their notable health care capacity in recent years.

In generally, anthocyanin shows red pigment in acidic conditions while blue pigment appears in alkaline conditions (Coutinho et al., 2015). In the last two decades, efforts have been made to decipher the biosynthesis of anthocyanins in many plants from biochemical, physiological, or molecular genetic perspectives. In flowering plants, anthocyanins are synthesized *via* the flavonoid pathway under the control of multiple enzyme and regulatory genes (Jaakola, 2013). In *C. sinensis*, with the genome assembling, the key gene families involved in anthocyanin biosynthesis, such as chalcone synthase (*CHS*), chalcone isomerase (*CHI*), dihydroflavonol reductase (*DFR*), leucoanthocyanidin reductase (*LAR*), and anthocyanidin reductase (*ANR*) were identified (Wei et al., 2018). Furthermore, anthocyanin late biosynthetic genes, such as flavonoid 3-hydroxylase (*F3H*), flavonoid 3′;- hydroxylase (*F3′;H*), flavonoid 3′; 5′;- hydroxylase (*F3′;5′;H*), anthocyanidin synthase (*ANS*) and UDP glucose-flavonoid 3-o-glcosyl-transferase (*UFGT*), exhibit the specific expression in purple-leaf tea palnts (Rothenberg et al., 2019; Chen et al., 2020). Meanwhile, transcriptional regulation of structural genes appears to be a major factor contributed to the anthocyanin biosynthesis in plants. To date, a MYB-bHLH-WD40 (MBW) complex, which is assembled through MYB transcription factors (TFs), basic helix-loop-helix (bHLH) TFs, and WD-repeat proteins, is responsible for anthocyanin biosynthesis (Ramsay and Glover, 2005). In ‘Zijuan’ tea plants, A R2R3-MYB TF CsAN1 interacts with bHLH TFs (CsGL3 and CsEGL3), and recruits a WD-repeat protein CsTTG1 to form the MBW complex which largely determine the anthocyanin accumulation in the foliage of ‘Zijuan’ cultivar with purple leaf (Sun et al., 2016). Another R2R3-MYB TF CsMYB2, not the same as CsAN1, positively regulates the expression level of *CsF3′;H* (Wang et al., 2018). Furthermore, other TFs, such as MADS, WRKY, NAC, and bZIP, have also been shown to participate in regulatory control of anthocyanin biosynthesis in pear, apple, and bilberry (Jaakola et al., 2010; An et al., 2017; Sun et al., 2019; Li et al., 2020a). However, the genetic rules of anthocyanin accumulation in tea plants have not been well deciphered.

Drinking anthocyanin-rich tea promotes simultaneous ingestion of catechins and anthocyanins. Therefore, breeding abundant anthocyanin-rich tea cultivars was carried out. ‘Zijuan’ (ZJ) confers an abnormal accumulation of anthocyanin, resulting in a colored phenotype that has a claret-purple young foliage. In this study, we first set up a hybrid combination of ‘ZJ’ and green-leaf cultivar ‘Fudingdabaicha (FD)’, as well as found that anthocyanin accumulation occurred significant segregation in F1 generation plants. Widely targeted metabolomics technology was first used to investigate the contents and types of anthocyanins, and results showed that cyanidin-3-*O*-galactoside, delphinidin-3-*O*-galactoside and petunidin-3-*O*-galactoside were the major factors which directly led to the color variation between parents and F1 plants. Transcriptomic analyses suggested the significant upregulation of an anthocyanidin synthase gene (*CsANS1*) in anthocyanin-rich tea plants and cloning and alignment further suggested that a deletion mutant of *CsAN1*, a MYB family gene positively regulated the expression of *CsANS1*, potentially lead to drawback of anthocyanins accumulation in anthocyanins-lacking tea plants.

## Materials and methods

### Plant materials

The claret-colored leaf cultivar ‘ZJ’ (*C. sinensis* var. *assamica*) and green-leaf cultivar ‘FD’ (*C. sinensis* var. *sinensis*) were used as female and male parents to cross-fertilize, respectively. The F1 generation plants, including ‘YG’ (yellow-green leaf), ‘GP’ (mauve leaf), ‘RP’ (claret-colored leaf) and ‘BP’ (dark-purple leaf) were obtained. All materials were planted in the Changsha region (Changsha, Hunan, China). All materials were grown under the natural environmental conditions. The second tender leaf under the bud (100 g) of parents and progenies were collected and frozen in liquid nitrogen. Three biological replicates for every sample used. All samples were ground and stored at -80°C until used.

### Anthocyanin extraction

For anthocyanin extraction, 0.05 g powder was accurately weighted and extracted with 500 μL methanol/water/hydrochloric acid (500:500:1, V/V/V). All extracts were vortexed for 5 min and ultrasound for more 5 min and centrifuged at 12, 000 rpm under 4°C for 3 min. The sediments were re-extracted according to the above steps again under the same conditions, respectively. The supernatants were collected, and filtrated through a membrane filter (0.22 μm, Anpel) before LC-MS/MS analysis. For determination of total anthocyanins, 1 g sample was quickly sliced and extracted with 15 ml HCl-methanol (0.15% HCl: 95%methanol, v: v=15: 85) for 4 h. The extract was centrifuged and its absorbance was determined at 530, 620 and 650 nm, respectively. The anthocyanin content measurement was based on the formula: ΔA/ml = (A_530_-A_620_) - 0.1 (A_650_-A_620_) (Zheng and Tian, 2006).

### ESI-Q TRAP-MS/MS analyses

The sample extracts were detected by LC-ESI-MS/MS system (HPLC, Shim-pack UFLC SHIMADZU CBM30A system, www.shimadzu.com.cn/; MS, Applied Biosystems 4500 Q TRAP, http://www.appliedbiosystems.com.cn/). The liquid chromatography analytical conditions were as follows: 5 µL of sample was injected into a Waters ACQUITY UPLC HSS T3 C18 (1.8 µm, 2.1 mm*100 mm). The HPLC mobile phase was 0.04% acetic acid in acetonitrile (solvent B) versus 0.04% acetic acid in Milli-Q water (solvent A). Separation was achieved with the following gradients: starting with 5% solvent B, raising to 95% B in 11 min, maintaining at 95% B for 1 min, dropped quickly to 5% B within 0.1 min and maintaining at 5% B for 3 min. The flow rate was 0.40 mL/min at a temperature of 40°C.

Linear ion trap (LIT) and triple quadrupole (QQQ) scans were acquired on a triple quadrupole-linear ion trap mass spectrometer (API 4500 Q TRAP LC/MS/MS System; Boston, USA) equipped with an ESI Turbo Ion Spray interface, operating in both positive and negative ion model and controlled by Analyst 1.6.3 software (AB Sciex, Singapore). The ESI source operation parameters were as follows: ion source, turbo spray; source temperature, 550°C; ion spray voltage (IS), 5500 V; and ion source gas I (GSI), gas II (GSII), curtain gas (CUR) set to 55, 60 and 25 psi, respectively. The collision activated dissociation (CAD) was set at “high”. Instrument tuning and mass calibration were performed with 10 and 100 μmol/L polypropylene glycol solutions in QQQ and LIT modes, respectively. QQQ scans were acquired *via* multiple reaction monitoring (MRM) experiments with collision gas (nitrogen) set to 5 psi. Declustering potential (DP) and collision energy (CE) were optimized for individual MRM transitions. A specific set of MRM transitions was monitored for each period according to the metabolites eluted within the period (Zou et al., 2020).

### MS data and statistical analyses

MS data acquisition and processing were performed as described previously (Chen et al., 2013). The analyses of the primary and secondary MS data were performed based on the self-built database MWDB (Metware Biotechnology Co., Ltd. Wuhan, China). Metabolite quantification was accomplished with data acquired in MRM mode by QQQ-MS (Zou et al., 2020). Metabolites with a fold change ≥2 or a fold change ≤0.5 were identified as upregulated or downregulated.

### RNA extraction and RT-qPCR

Total RNA from each frozen sample was extracted by modified CTAB method (Gambino et al., 2008). RNA sample integrity was assessed by agarose gel electrophoresis and Agilent 2100 system (Agilent Technologies Inc., USA). The concentration of RNA was measured using NanoDrop 2000 spectrophotometer (Eppenforf, Germany). Total RNA (0.5 µg) was used to synthesize the first-strand cDNA with random primer according to the manufacturer’s instructions using a RevertAid First Strand cDNA Synthesis Kit (ThermoFisher, USA). The expression patterns of all candidates were verified by quantitative real-time PCR (RT-qPCR). RT-qPCR was conducted in CFX Real-Time PCR Detection System (Bio-Rad, USA) using the SYBR qPCR Mix (Vazyme, Nanjing, China). The 20 µL reactionmixture contained about 1 µL template cDNA, 0.2 µM of each forward and reversegene-specific primers and 10 µL SYBR. The relative expression levels were calculated using the 2^-ΔΔCT^ formula (Livak and Schmittgen, 2001). For gene cloning, the first-strand cDNA was synthesized by the oligo (d_T_)_18_ primer, and the full-length of candidate was cloned using Phanta Max Super-Fidelity DNA Polymerase (Vazyme, nanjing, China) according to the manufacture’s descriptions. All primers used in this study were presented in **Table S6**.

### Library preparation for transcriptome sequencing

The RNA sequencing libraries were generated were generated using NEBNext^®^ UltraTM RNA Library Prep Kit for Illumina^®^ (NEB, USA) following manufacturer’s recommendations. Briefly, mRNA was purified from total RNA using poly-T oligo attached magnetic beads. Fragmentation was carried out using divalent cations under elevated temperature in NEBNext First Strand Synthesis Reaction Buffer (5×). First strand cDNA was synthesized using random hexamer primer and M-MuLV Reverse Transcriptase (RNase H-). Second strand cDNA synthesis was subsequently performed using DNA Polymerase I and RNase H. Remaining overhangs were converted into blunt ends *via* exonuclease/polymerase activities. After adenylation of 3′; ends of DNA fragments, NEBNext Adaptor with hairpin loop structure were ligated to prepare for hybridization. In order to select cDNA fragments of preferentially 250~300 bp in length, the library fragments were purified with AMPure XP system (Beckman Coulter, Beverly, USA). Then 3 µl USER Enzyme (NEB, USA) was used with size-selected, adaptor-ligated cDNA at 37°C for 15 min followed by 5 min at 95°C before PCR. Then PCR was performed with Phusion High-Fidelity DNA polymerase, Universal PCR primers and Index (X) Primer. At last, PCR products were purified (AMPure XP system) and library quality was assessed on the Agilent Bioanalyzer 2100 system. The libraries were sequenced on an Illumina^®^HiSeq2500 platform and 125 bp/150 bp paired-end reads were generated.

### Gene functional annotation and expression analysis

The raw data were filtered by removing low quality reads and adaptors, and were changed into clean reads. the HISAT v2.1.0 package was used to construct the index, and mapped clean reads to the reference genome (http://tpdb.shengxin.ren/). The feature Counts v1.6.2 package was used to calculate the gene alignment, and then calculate the FPKM of each gene based on the gene length. The DESeq2 v1.22.1 was used to analyze the differential expression between the two groups, and the *p*-value was corrected using the Benjamini & Hochberg method. Gene function was annotated according to these databases: (NCBI non-redundant protein sequences (Nr); Clusters of Orthologous Groups of proteins (COG/KOG); Swiss PROT protein sequence database (Swissprot); Kyoto Encyclopedia of Genes and Genomes (KEGG); homologous protein family (Pfam) and Gene Ontology (GO).

### Statistical analysis

The standard deviation (SD) of the metabolite concentrations were calculated based on three biological replicates and technical replicates. SPSS 17.0 software was used to determine the significant differences by Duncun method with *p <*0.05.

## Results

### Anthocyanin accumulation variation in F1 plants

**Figure 1:**
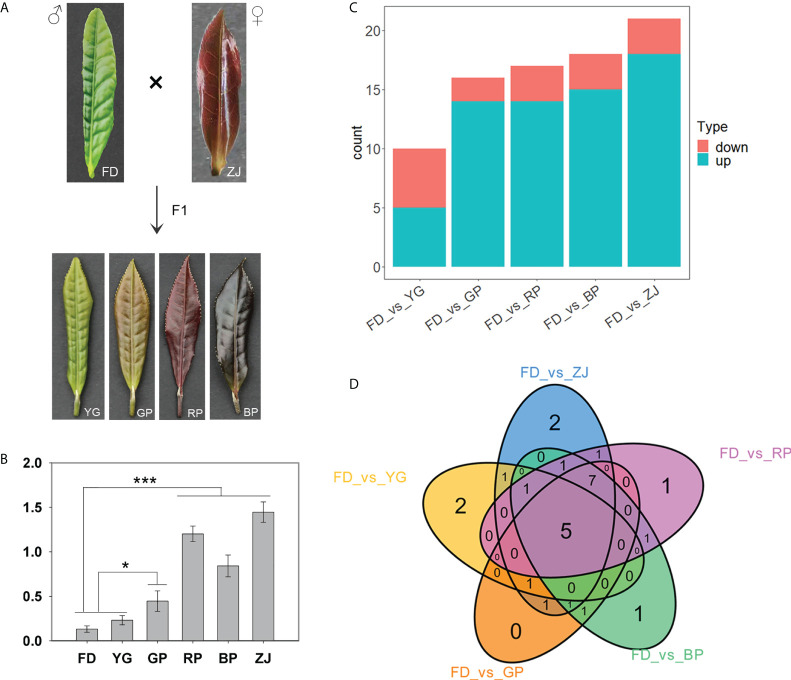
Metabolic profiling of the parents and F1 hybrid progenies. **(A)** The morphological phenotypes of the parents and F1 hybrid progenies; **(B)** The anthocyanin content of parents and hybrid progenies; **(C)** Differential metabolites in various groups; **(D)** Venn diagram of differential metabolites between 5 groups. “*”: *p <*0.05; “***”: *p <*0.001.

The claret-colored foliage cultivar ‘ZJ’ and green-foliage cultivar ‘FD’ were used as female and male parents, respectively, to generate F1 hybrid progenies. Among F1 offspring, significant differences were observed in the morphological phenotypes, especially concerning leaf color, including yellow-green leaf (‘YG’), mauve leaf (‘GP’), claret-colored leaf (‘RP’) and dark-purple leaf (‘BP’) (**Figure 1A**). To investigate overall anthocyanin differences among samples, the total anthocyanin content was determined. Those results showed that the anthocyanin content in ‘ZJ’ was the highest, and the anthocyanin content of ‘GP’, ‘RP’, and ‘BP’ were significantly higher than that in ‘FD’ and ‘YG’ (**Figure 1B**). Additionally, ‘RP’ is phenotypically similar in color with the parent ‘ZJ’. In *C. sinensis*, it has been proved that anthocyanin accumulation differences contribute to the color variation. Therefore, metabolic profiling was first performed to analyze between parents and progenies and to focus on anthocyanin contents. Metabolic profiling of the 6 samples using an UPLC-ESI-MS/MS system identified 57 metabolites (**Table S1**). According to the metabolome data, the metabolites identified include the following: 7 cyanidins, 13 delphinidins, 7 flavonoids, 4 malvidins, 8 pelargonidins, 13 peonidins and 5 procyanidins (**Table S1**). Comparing with the male parent, 10 differentially accumulated metabolites were detected between yellow-green leaf cultivar ‘YG’ and green-leaf cultivar ‘FD’, including 5 downregulated and 5 upregulated metabolites, respectively (**Figures 1C, D** and **Table S2**). Furthermore, comparing with the green-leaf cultivar ‘FD’, most of the differentially accumulated anthocyanins were significantly up-regulated in purple tea plants, including ‘GP’, ‘RP’, ‘BP’ and ‘ZJ’ (**Figures 1C, D** and **Table S2**). Those results suggested that, in *C. sinensis*, anthocyanin accumulation differences contributed to the color variation in F1 hybrid progenies.

### Main types of anthocyanidin accumulation in *C. sinensis*

**Figure 2:**
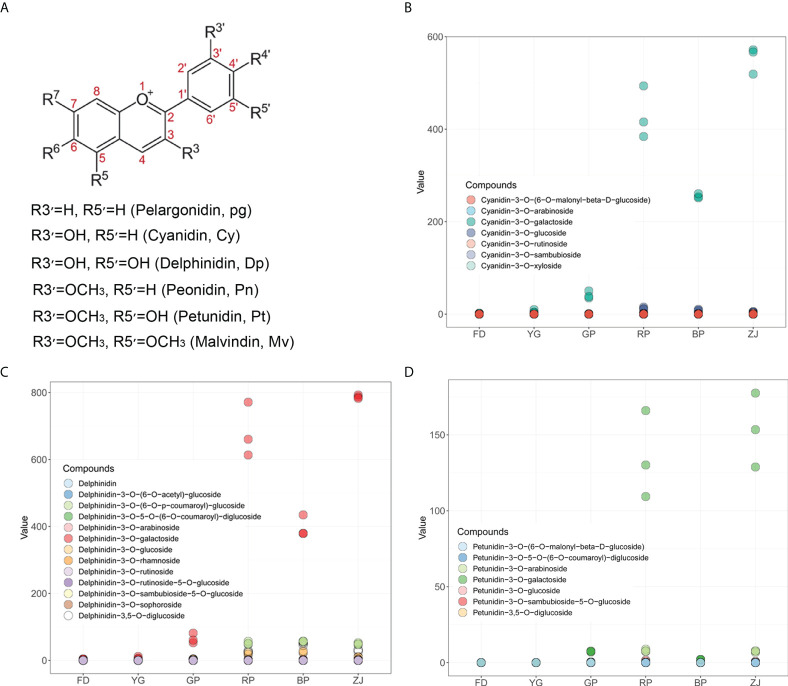
The main types of anthocyanins accumulation in *C. sinensis*. **(A)** The basic structure of anthocyanidins; **(B)** The accumulation variation of anthocyanins derived from Cy type in parents and F1 hybrid progenies; **(C)** The accumulation variation of anthocyanins derived from Dp type in parents and F1 hybrid progenies; **(D)** The accumulation variation of anthocyanins derived from Pt type in parents and F1 hybrid progenies.

Generally, anthocyanins structurally comprise an anthocyanidin aglycon bound to one or more sugar moieties. Six anthocyanidin types, namely cyanidin (Cy), delphinidin (Dp), pelargonidin (Pg), peonidin (Pn), petunidin (Pt), and malvidin (Mv), occur in colored plants (**Figure 2A**) (Jaakola, 2013). In order to investigate which types of anthocyanidin mainly accumulated in *C. sinensis*, the corrected peak area of each metabolite was calculated. Those results showed that Cy, Dp and Pt were the main types anthocyanidin aglycon accumulated in tea plants (**Figures 2B–D**). Though Mv, Pg and Pn were characterized and up-regulated accumulation in ‘RP’ and ‘ZJ’, the peak area of each metabolite was much lower (**Figures S1A, B, E**), suggesting that the contents of Mv, Pg and Pn were negligible. In addition, cyanidin-3-*O*-galactoside was the predominant anthocyanin and significantly up-regulated in tea plants with purple-leaf (**Figure 2B**). In the type of Dp, delphinidin-3-*O*-galactoside was the remarkable anthocyanin and significantly up-regulated in tea plants with purple-leaf, which was similar with the accumulation pattern of Cy (**Figure 2C**). Furthermore, petunidin-3-*O*-galactoside was the major component of the Pt type, which is notably accumulated in ‘RP’ and ‘ZJ’ with claret-colored leaf and, however, hardly detected in ‘BP’ with dark-purple leaf (**Figure 2D**). Though some flavonoids and procyanidins were characterized, however, there were no obvious accumulation differences (**Figures S1C, D**). Taken together, accumulation differences of cyanidin-3-*O*-galactoside, delphinidin-3-*O*-galactoside and petunidin-3-*O*-galactoside were the major factors which directly led to the color variation between parents and F1 hybrid progenies.

### Identification of differentially expressed genes using transcriptomic analyses

To investigate the mechanism which led to the anthocyanin various between parents and F1 groups, an RNA-seq library with the total RNA of tea plants leaf samples were constructed and a total of 43.76-57.21 million raw reads were produced by using the Illumina^®^HiSeq2500 platform (**Table S3**). After processing raw reads, 6.32-8.35 G clean base with a Q30 percentage of 92.07%-93.54% and GC content percentage of 43.71%-44.41% were obtained and been available for analyses (**Table S3**). All the reads were successfully annotated through alignment to the reference genome with percentage of 85.54%-87.24% (**Table S3**).

**Figure 3:**
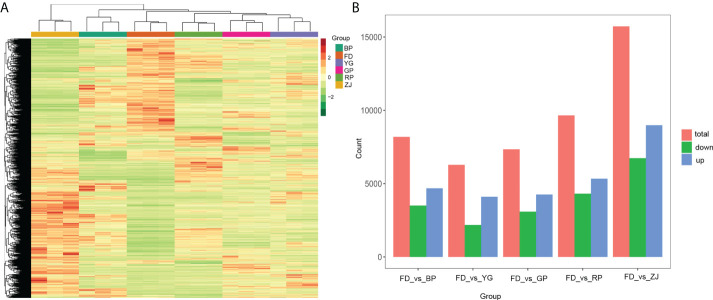
Differentially expressed genes (DEGs) at various sample groups. **(A)** The cluster analysis of all DEGs; **(B)** DEGs among ‘FD’_*vs*_’BP’, ‘FD’_*vs*_’YG’, ‘FD’_*vs*_’GP’, ‘FD’_*vs*_’RP’ and ‘FD’_*vs*_’ZJ’.

The differentially expressed genes (DEGs) were compared in all samples. In total, 21, 271 DEGs were identified, including a great deal of putative novel transcripts (**Table S4**). Cluster analysis showed that there was a high degree of consistency among biological duplications of DEGs (**Figure 3A**). In addition, the results showed that the DEGs patterns were remarkably opposite between the two parents (‘FD’ and ‘ZJ’) (**Figure 3A**). The number of DEGs had very high variance among different groups. The largest number of DEGs was found between ‘FD’ and ‘ZJ’ and the least number of DEGs was found between ‘FD’ and ‘YG’, which is the consistent with morphological phenotypes of the parents and F1 hybrid progenies (**Figure 3B**).

### Differential genes expression in anthocyanin biosynthetic pathway

**Figure 4:**
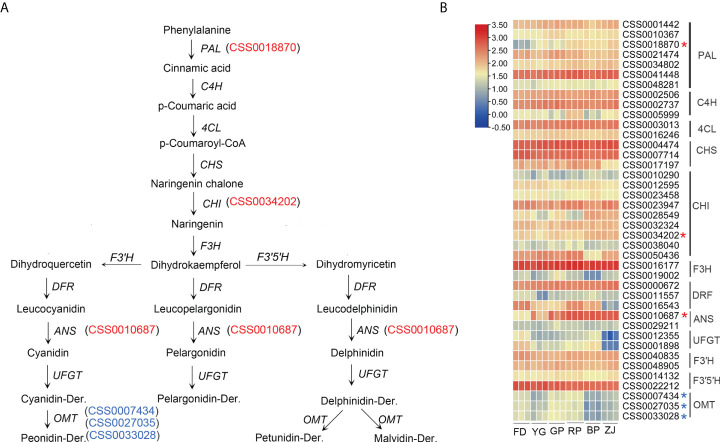
Transcript profiling of genes in the anthocyanin biosynthetic pathway. **(A)** anthocyanin biosynthetic pathway; **(B)** Heat map presentation of the expression patterns of anthocyanin-related genes The up-regulated genes in purple tea plants were marked by red "*", and the OMT genes were marked by blue "*" in **(B)**.

**Figure 5:**
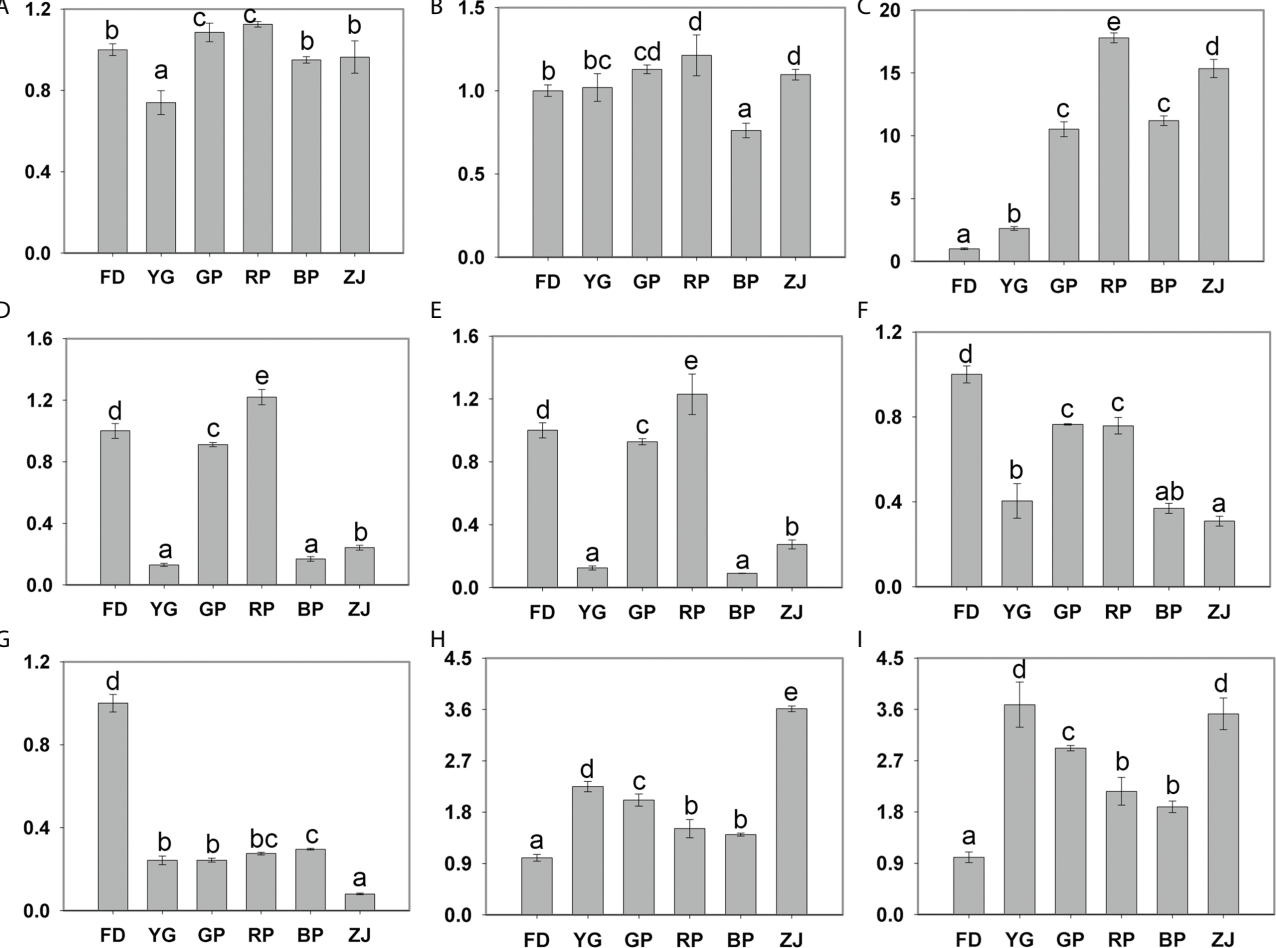
Expression of differentially expressed genes by RT-qPCR validation. **(A)** *PAL* (CSS0018870); **(B)** *CHI* (CSS0034202); **(C)** *ANS* (CSS0010687, *CsANS1*); **(D)** *OMT* (CSS0007434); **(E)** *OMT* (CSS0027035); **(F)** *OMT* (CSS0033028); **(G)** MYB family gene (CSS0015793); **(H)** MADS family gene (CSS0016295); **(I)** WRKY family gene (CSS0024910). Significant difference at p < 0.05 is indicated by different letters (a–e) above the columns.

Anthocyanins are synthesized by the phenylpropanoid pathway through enzymatical shift of phenylalanine to 4-coumarol-CoA, resulting in entering the anthocyanin biosynthesis pathway (**Figure 4A**). In this study, enrichment analyses showed that 40 genes were involved in the anthocyanin biosynthesis pathway, which were differentially expressed between parents and F1 hybrid progenies (**Figure 4B**). The first step synthetic reaction was the phenylalanine de-amination reaction catalyzed by the phenylalanine ammonia-lyase (PLA) which is encoded by *PLA* gene. In the study, a *PAL* (CSS0018870) was up-regulation with the anthocyanin accumulation in ‘GP’, ‘RP’, ‘BP’ and ‘ZJ’ (**Figure 4B**). It was suggested that chalcone synthase played an important role in formation of anthocyanin precursors. In present study, nine *CHI* genes were identified by functional annotation, and among them, one *CHI* (CSS0034202) were up-expression with anthocyanin accumulation (**Figure 4B**). However, the expression pattern of *PAL* and *CHI* were not consistent with the RT-qPCR verification (**Figures 5A, B**). Two key *ANS* genes (CSS0010687 and CS0029211) were found in our dataset, notably, CSS0010687 (named *CsANS1*) was remarkable up-expression in the anthocyanins-rich cultivar (**Figure 4B**). Additionally, previous researches have been suggested that *O*-methyltransferase (OMT) modifies accumulation of methylated anthocyanin in plants (Gomez Roldan et al., 2014). In present study, three *OMT* genes (CSS0007434, CSS0027035 and CSS0033028) were identified and down-regulated in ‘BP’, ‘ZJ’, and ‘YG’ with dark-purple leaf (**Figure 4B**). Those results showed that the structural genes related to anthocyanins biosynthesis were differentially expressed.

### Verification of anthocyanin-related genes by using RT-qPCR

To further verify the RNA-seq expression profile data, RT-qPCR assays were performed based on the six differentially expressed structural genes, including one *PAL* (CSS0018870), one *CHI* (CSS0034202), one *CsANS1* (CSS0010687), three *OMT* (CSS0007434, CSS0027035 and CSS0033028) and three random selected TF genes (CSS0015793, CSS0016295 and CSS0024910). With respect to the results, all DEGs were found to be consistent with RNA-Seq data except *PAL* (CSS0018870) and *CHI* (CSS0034202) (**Figure 5**). Notably, the expression pattern of *CsANS1* (CSS0010687) was significantly up-regulated in colored leaves of tea cultivars (‘GP’, ‘RP’, ‘BP’ and ‘ZJ’) (**Figure 5C**). In addition, the expression pattern of CSS0015793 and CSS0016295 were consistent with the transcriptomic analyses and that of CSS0024910 is inconsistent (**Figures 5G–I** and **Table S4**). Taken together, the remarkable up-regulation of *CsANS1* directly contributed to the color variation through catalyzing anthocyanin biosynthesis in purple-leaf tea cultivars.

### Identification of MBW complex members involved in the anthocyanin biosynthetic regulation

**Figure 6:**
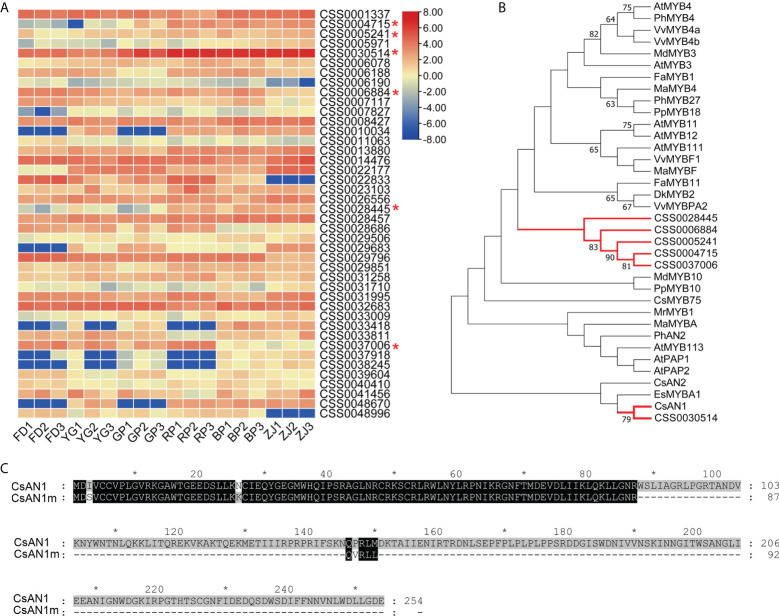
Identification of the MYB family members. **(A)** Heat map description of the expression patterns of MYB family members in parents and F1 hybrid progenies (the candidates of MYB family gene were marked by red "*"); **(B)** Phylogenetic analysis of candidate MYB family members; **(C)** Sequences alignment of *CsAN1* (Gene bank accession: KU745295) and putative protein encoded by *CsAN1m* (CSS0030514) (the gray ‘*’ means the numbers spaced by ten).

In order to identify the TFs involved in the anthocyanin-related regulation, all TFs detected in the RNA_seq data were filtrated based on the references of *C. sinensis*. A total of 2903 TFs were expressed in all samples, including MYB family, bHLH family, WD40 family, and WRKY family (**Table S5**). We first focused on the MBW complex which belongs to the key components regulating structural genes related to anthocyanin biosynthesis and found that the expression patterns of six genes (CSS0004715, CSS0005241, CSS0030514, CSS0006884, CSS0028445, CSS0037006) encoding MYB family members were associated with anthocyanin accumulation (**Figure 6A**). The expression of bHLH and WD40 family genes were also analyzed and no significant expression differences were found (**Figure S2**). Phylogenetic analyses found that all the MYB family members were grouped into the common clade except CSS0030514. Notably, the CSS0030514 (named *CsAN1m* in this study), homologous with *AtMYB113* in Arabidopsis, had a much higher FPKM value in the RNA_seq data and significantly up-regulated with the increase of anthocyanin content (**Figure 6A**). Furthermore, *CsAN1m* was confidently clustered with *CsAN1* which has been proven to be a MYB family gene responsible for positive regulation of the anthocyanin-related genes in ‘Zijuan’ tea plants (**Figure 6B**). However, *CsAN1m* was annotated in reference genome and only encode 92 residues with almost identical N-terminal sequences with CsAN1 (**Figure 6C**). Therefore, it is putative that the *CsAN1m* is the ortholog of *CsAN1* with truncated mutation and probably lost regulatory functions.

### *CsAN1* responsible for key regulation of anthocyanin accumulation

**Figure 7:**
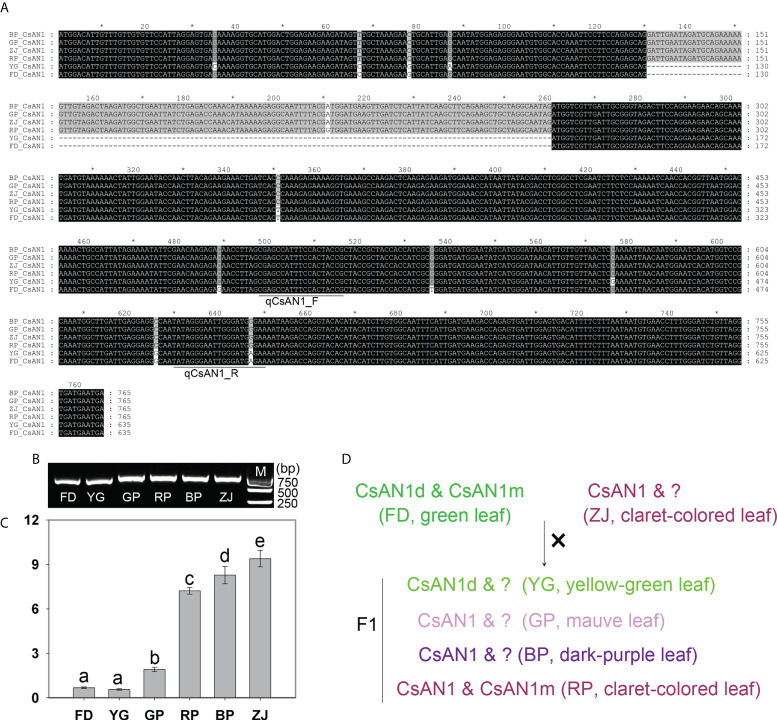
Cloning and expression analysis of *CsAN1.* **(A)** The sequence alignment of *CsAN1* in the parents and F1 hybrids (the gray "*" means the numbers spaced by ten); **(B)** RT-PCR analysis of *CsAN1* in the parents and F1 hybrids; **(C)** the expression pattern of *CsAN1* in the parents and F1 hybrids; **(D)** The putative regulatoary model of anthocyanin accumulation in F1 hybrids. qCsAN1_F and qCsAN1_R was the primer pair for RT-qPCR detection. “?” represented the unknown *CsAN1* ortholog. Significant difference at p < 0.05 is indicated by different letters (a-e) above the columns.

To verify the existence and expression of *CsAN1* and *CsAN1m*, we cloned and sequenced from all tested samples. Interestingly, all *CsAN1* were successfully cloned from parents and F1 plants, while a fragment with 160-bp length was delated in green-leaf ‘FD’ and yellow-green leaf ‘YG’, named *CsAN1d* in this study (**Figure 7A, B**). We also designed the specific primer pair to cloned the *CsAN1m*, however, we just obtained it from green-leaf ‘FD’ and claret-colored leaf ‘RP’ (**Figure S3**). In addition, the expression pattern of *CsAN1* was examined in tested cultivars, in consistent with prediction, the expression level was significantly up-regulated in anthocyanin-rich tea plants (**Figure 7C**). Taken together, it is putative that non-functional *CsAN1* mutant (*CsAN1d* and *CsAN1m*) leads to the drawback of anthocyanin biosynthesis in ‘FD’ cultivar. While in ‘ZJ’ cultivar, a normal *CsAN1* was sufficient to positively regulates the anthocyanin accumulation. And in F1 hybrid plants, *CsAN1* mutant and normal *CsAN1* were separated, resulting in anthocyanin content differences (**Figure 7D**).

## Discussion

Anthocyanins, belonging to flavonoids, were the key pigments for determining tea plants coloration, and they are synthesized by the phenylpropane metabolic pathway (Saito et al., 2011). Generally, anthocyanin structures are a composite of six types common anthocyanidin aglycon. ‘ZJ’ variety, an anthocyanin-rich tea cultivar, has been widely cultivated in the Yunnan province of China, as well as the amount of anthocyanins in ‘ZJ’ cultivar was up to 707 ± 28 μg/g of dry weight (Jiang et al., 2013). At least four main anthocyanins were successfully identified in previous study, including delphinidin-3-*O*-galactoside, cyanidin-3-*O*-D-galactoside, delphinidin-3-*O*-D-(6-(E)-pcoumaroyl) galactopyranoside and cyanidin-3-*O*-D-(6-(E)-p-coumaroyl) galactopyranoside (Jiang et al., 2013). In our study, six common members were also characterized by the widely targeted metabolomic analyses. Those results showed that cyanidin-3-*O*-galactoside, delphinidin-3-*O*-galactoside and petunidin-3-*O*-galactoside mainly contribute to the anthocyanin types and content, except the dark-purple ‘BP’ cultivar (**Figures 1A** and **2**). In ‘BP’ cultivar, the petunidin-3-*O*-galactoside is scarcely accumulated and the accumulation level of cyanidin-3-*O*-galactoside and delphinidin-3-*O*-galactoside are much lower compared with deep-colored ‘RP’ and ‘ZJ’ varieties (**Figure 2**). Previous studies suggested that the peonidin-derivates (peonidin-der.) are methylated by the OMT. And more, the OMT also catalyzes the methylation reaction of delphinidin-derivates (delphinidin-der.) and transformed into petunidin and malvidin derivates (Gomez Roldanet al., 2014). In this study, three *OMT* were identified and have similar expression pattern with lower expression level in ‘BP’ and ‘YG’ comparing with ‘RP’ and ‘ZJ’ (**Figures 4B**, **5D–F**). So, it is speculated that this is the reason caused the loss of petunidin-der. biosynthesis (**Figure 2**). Transcriptomic analysis suggested that the expression levels of the *OMT* were down-regulated in ‘YG’, ‘BP’, and ‘ZJ’, and that of them were higher in ‘FD’, ‘GP’, and ‘RP’ by transcriptomic analyses (**Figure 4A**), which is consistent with the RT-qPCR results (Figure D, E, F). However, the higher content Pt in ‘ZJ’, potentially own to the up-expressed of *CsANS1* (CSS0010687) and the excessive accumulation of Dp (**Figures 2C** and **5C**). Conversely, though the *OMT* were higher expression level in ‘FD’, the substrate accumulation for the methylation reaction was much less. Additionally, anthocyanin components also play a key role in leaf color. Cy, Pn, and Pt are the main anthocyanidins in pink and red leaf and dark red leaf, while Dp and Mv anthocyanidins are generally higher in purple black, blue purple, blue black and other dark leaf (Khoo et al., 2017). Therefore, the lower accumulation level of Cy and Pt potentially lead to the dark-purple leaf of ‘BP’ cultivar (**Figure 2B**).

Previous studies have certified that anthocyanin biosynthesis is mediated by multiple enzymes encoded by early structural genes (*PAL*, *C4H*, *4CL*, *CHS*, *CHI*, and *F3H*) and late anthocyanin-specific biosynthesis genes (*F3′;H*, *F3′;5′;H*, *DFR*, *ANS*, *UFGT* and *OMT*) (Jaakola, 2013). In the present study, the expression pattern of all genes related to anthocyanin synthesis pathway were verified by the transcriptome analysis. The results suggested that the expression levels of *PAL*, *CHI* and *ANS* in purple cultivars were higher than those in green cultivars (**Figure 4B**). RT-qPCR was performed to verified the expression of those genes (**Figures 5A–C**). Though a previous study suggested that the *PAL* expression was upregulated in purple leaves of the ‘ZJ’ cultivar (Li et al., 2016), while the expression level of *PLA* was not obvious in parents and F1 hybrid plants by RT-qPCR analysis (**Figure 5A**). *CHI* is another important enzyme-coding gene in anthocyanin biosynthesis pathway, the RT-qPCR analysis showed that its expression difference was also insignificant (**Figure 5B**). Generally, transcriptome analysis is used for large-scale screening to reflect the overall gene expression trend of the sample, but it cannot guarantee that the change trend of every gene is consistent with that of RT-qPCR. ANS is one of the key enzymes that occur late in the anthocyanin synthesis pathway, and it can catalyze the conversion of leucodelphinidin, leucopelargonidin, and leucocyanidin to anthocyanins (Saito et al., 2002). We found that the expression level of *CsANS1* in purple tea plants is considerably higher, which is consistent with the accumulation of anthocyanins content (**Figures 1B** and **5C**). In addition, we found that the gene expression level of *CsANS1* is slightly inconsistent with the anthocyanin content in ‘RP’ and ‘ZJ’, in which the expression of *CsANS1* was up-regulated in ‘RP’ comparing with ‘ZJ’, but the anthocyanins content was lower in ‘ZJ’ (**Figures 1B** and **5C**). In fact, the anthocyanin biosynthesis is regulated by different factors and even some negative regulatory factors (Sun et al., 2019; Li et al., 2020a). Therefore, it is reasonably putative that other regulators exclusively contribute to the anthocyanin content in tea cultivars. Thus, we also speculated that the high expression of *CsANS1* is the key factor contributed to the color variation through catalyzing anthocyanin biosynthesis in purple-leaf tea cultivars.

Meanwhile, anthocyanin-related biosynthesis genes are regulated by the MYB-bHLH-WD40 (MBW) protein complex. In ‘Zijuan’ tree, the MBW complex regulates anthocyanin accumulation by upregulation mRNA expression of *F3H*, *DFR* and *ANS* (Sunet et al., 2016). In the present study, the MBW members were identified based on the transcriptomic analyses, respectively (**Figure 6A** and **Figure S2**). We found that a MYB family member, *CsAN1m* (CSS0030514), was significantly up-regulated with the expression of *CsANS1* and anthocyanins accumulation (**Figure 7C**). Interestingly, CsAN1m and CsAN1 have highly identical N-terminal sequence (**Figure 6C**). It has been proven that the CsAN1, a MYB family member, can assembles into MBW complex and positively activates the expression of *CsANS1*, increasing the accumulation of anthocyanins in ‘ZJ’ cultivar (Sun et al., 2016). So, we speculated that the *CsAN1m* potentially mutated from *CsAN1* during evolution, leading to the functional deficiency. Furthermore, the *CsAN1* was cloned in all tested samples, and found that the *CsAN1* has a 160-bp deletion mutation (*CsAN1d*) in anthocyanins-lacking ‘FD’ and ‘YG’. In fact, MYB TF mutation was reportedly involved in the regulation of anthocyanin biosynthesis. In grapevine, *VvMYBA1* and *VvMYBA2* are the switch genes for regulation of anthocyanin in the berry skin. The *VvMYBA1* was shown not to be transcribed in white berries because of the insertion of a retrotransposon in the promoter, while *VvMYBA2* is inactivated by two non-conservative mutations (Walker et al., 2007). Therefore, the function inactivation of the regulatory gene *CsAN1* is responsible for the inability of anthocyanins accumulation in ‘FD’, and this trait is heritable in progenies through hybridization (**Figure 7D**). Notably, a functional *CsAN1* was cloned, while the expression level was lower in ‘GP’, we assumed additional regulatory factors are involved in the regulation of anthocyanin biosynthesis and accumulation. From the previous results, other transcription factors (TFs), such as NAC, WRKY, bZIP, MADS and ERF, have also been proven to play a role in regulating anthocyanin biosynthesis (An et al., 2017; Sun et al., 2019; Li et al., 2020a; Yi et al., 2021).

## Conclusion

In this study, the claret-colored leaf cultivar ‘ZJ’ and green-leaf cultivar ‘FD’ were first used as parents to generated F1 hybrid progenies. Anthocyanins accumulation differences were obviously observed between parents and offspring. Metabolic analyses showed that cyanidin-3-*O*-galactoside, delphinidin-3-*O*-galactoside and petunidin-3-*O*-galactoside mainly contribute to the anthocyanin content in colored tea plants. Transcriptomic analyses indicated that the up-regulated expression of anthocyanin synthesis gene, *CsANS1*, potentially served as the one of the key factors contributed to the anthocyanin accumulation in colored tea plants. And much higher expression of a MYB gene, *CsAN1*, is responsible for the anthocyanin accumulation in colored tea plants by positive regulation the expression of *CsANS1*.

## Data availability statement

The data presented in the study are deposited in the NCBI repository, accession number PRJNA820473.

## Author contributions

FH was responsible for the metabolic and transcriptomic analyses, and manuscript preparation. JD performed the hybrid assays. YuL performed the RT-qPCR and constructed discussions. YK was responsible for measurement of anthocyanin content. YiL and DD cloned the genes. YC prepared some figures with R scripts. SL was responsible for experiment design and reviewed the manuscript. All authors contributed to the article and approved the submitted version.

## Funding

Natural Science Foundation of Hunan Province (2020JJ5276); Hunan Provincial Seed Industry Innovation Project (2021NK1008); Hunan Agricultural Innovation Fund (2022CX34).

## Conflict of interest

The authors declare that the research was conducted in the absence of any commercial or financial relationships that could be construed as a potential conflict of interest.

## Publisher’s note

All claims expressed in this article are solely those of the authors and do not necessarily represent those of their affiliated organizations, or those of the publisher, the editors and the reviewers. Any product that may be evaluated in this article, or claim that may be made by its manufacturer, is not guaranteed or endorsed by the publisher.

## Supplementary material

Supplementary Figure 1 The less abundant anthocyanins accumulation in *C. sinensis*. **(A)** The accumulation variation of anthocyanins derived from malvidin type in parents and F1 hybrid progenies; **(B)** The accumulation variation of anthocyanins derived from pelargonidin type in parents and F1 hybrid progenies; **(C)** The accumulation variation of flavonoid in parents and F1 hybrid progenies; **(D)** The accumulation variation of procyanidins in parents and F1 hybrid progenies; **(E)** The accumulation variation of anthocyanins derived from peonidin type in parents and F1 hybrid progenies.

Supplementary Figure 2 Heat map description of the expression patterns of bHLH **(A)** and WD40 **(B)** family members in parents and F1 hybrid progenies.

Supplementary Figure 3 Cloning of *CsAN1m* from ‘FD’ and ‘RP’.
