# Supplementary figures and images for: Metabolomic and transcriptomic analyses reveal a MYB gene, CsAN1, involved in anthocyanins accumulation separation in F1 between ‘Zijuan’ (Camellia sinensis var. assamica) and ‘Fudingdabaicha’ (C. sinensis var. sinensis) tea plants
Source: Front Plant Sci. 2022 Sep 21;13:1008588. doi: 10.3389/fpls.2022.1008588 (PMC9532865; doi:10.3389/fpls.2022.1008588)

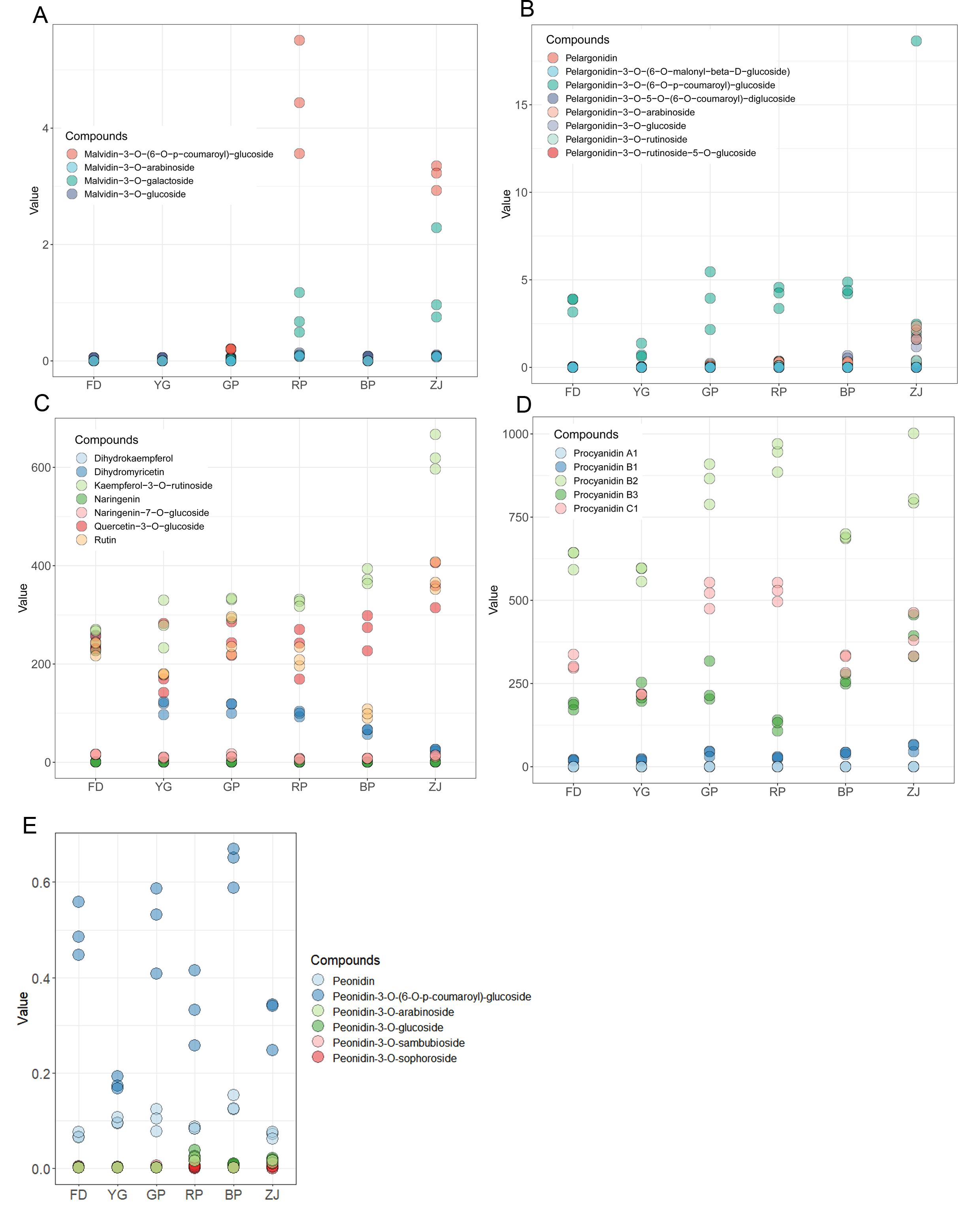

Supplement: Supplementary Figure 1 — The less abundant anthocyanins accumulation in C. sinensis. (A) The accumulation variation of anthocyanins derived from malvidin type in parents and F1 hybrid progenies; (B) The accumulation variation of anthocyanins derived from pelargonidin type in parents and F1 hybrid progenies; (C) The accumulation variation of flavonoid in parents and F1 hybrid progenies; (D) The accumulation variation of procyanidins in parents and F1 hybrid progenies; (E) The accumulation variation of anthocyanins derived from peonidin type in parents and F1 hybrid progenies. [file Image_1.tif]

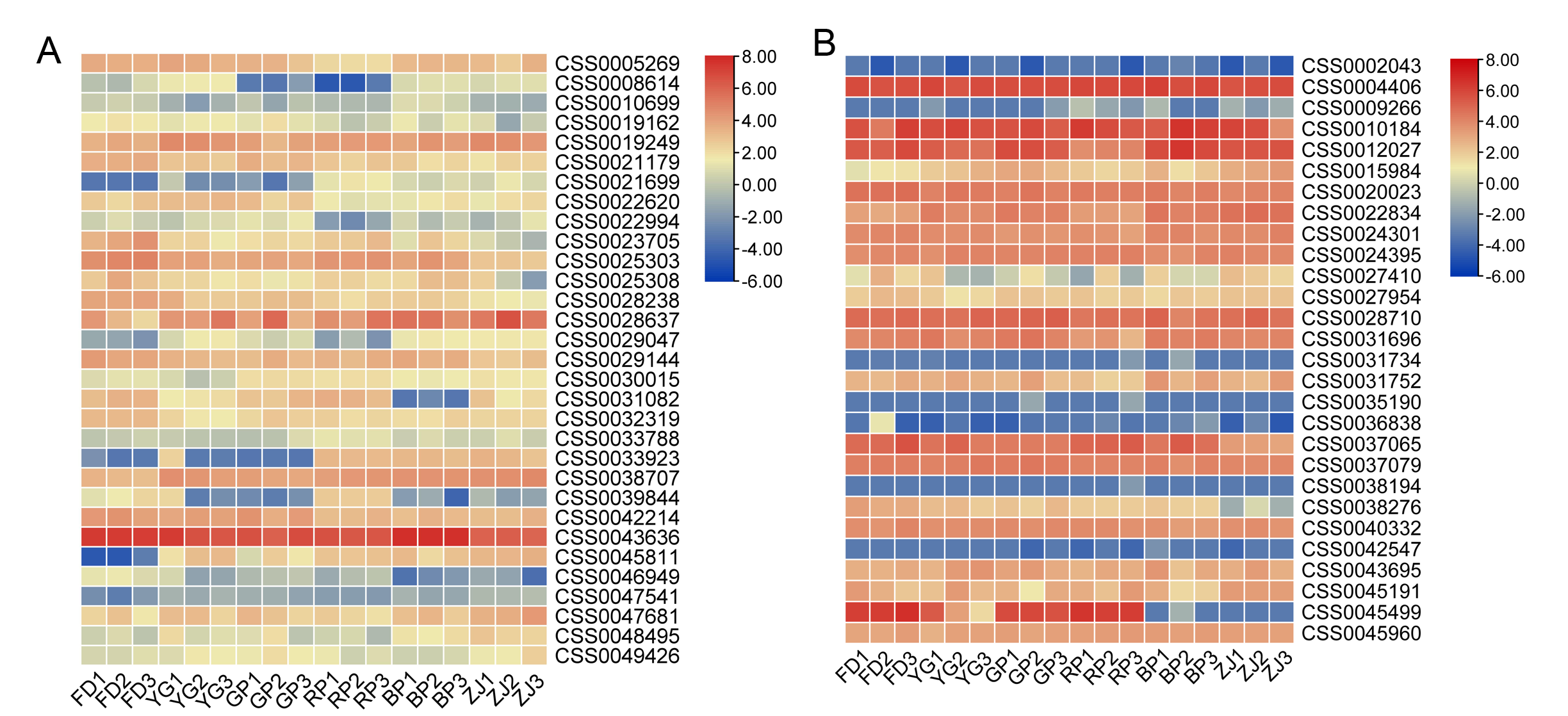

Supplement: Supplementary Figure 2 — Heat map description of the expression patterns of bHLH (A) and WD40 (B) family members in parents and F1 hybrid progenies. [file Image_2.tif]

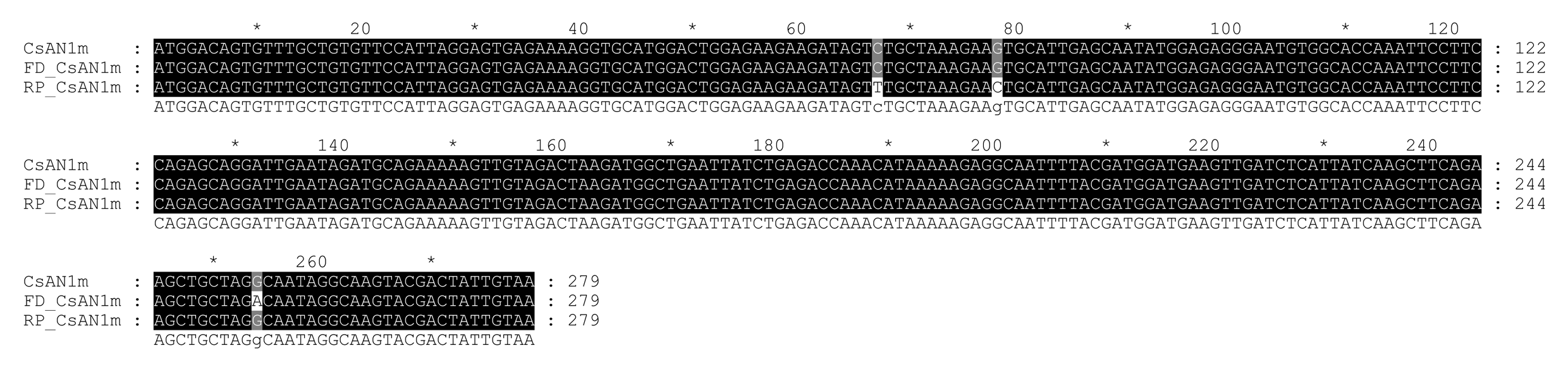

Supplement: Supplementary Figure 3 — Cloning of CsAN1m from ‘FD’ and ‘RP’. [file Image_3.tif]
